# Resuscitation of the Apparently Drowned

**Published:** 1890-12

**Authors:** 


ARTICLE IV.

### RESUSCITATION OF THE APPARENTLY DROWNED.

The *Medical Record*, Nov. 8, 1890, says: For the revival of persons who have been nearly drowned, two chief methods (with numerous slight modifications of each) are in use: the "Sylvester" and the "Marshall Hall." In 1862, a committee appointed by the Royal Medical and Chirurgical Society of London, to consider the rival claims of the two methods, reported in favor of the method of Sylvester, on the ground that the amount of air introduced into healthy lungs by this method was double that introduced by the method of Marshall Hall. The committee recommended that the body should be placed at an angle of thirty degrees, face downward, with the head lower than the feet, the mouth being open and the tongue drawn forward. The escape of fluids might be assisted by slight pressure on the back. After a few seconds the body should be laid back downward with a cushion under the shoulders, the tongue hanging out of the mouth. About fourteen times a minute the arms should be drawn above the head and then lowered and pressed, by the elbows, against the chest.

In the Transactions of the above-mentioned Society for 1889, Dr. Bowles criticises very earnestly the conclusions of the committee, which had been endorsed by the Society. The committee based its report upon experiments made with the *empty* lungs of animals. The physician, however, has to deal with human beings whose lungs are filled with fluid, mixed with air in a blood-stained froth. Under these conditions the most useful method must provide not only for the inspiration of air, but also for the escape of this frothy liquid from the deeper air passages. The sudden introduction of a large quantity of air by the

Sylvester method is injurious, because at the same time the froth is drawn deeply into the finest tubes, which before contained a little air. By the Marshall Hall method, however, the air is introduced gently and in small quantities, and the froth is not drawn in more deeply, but is gradually expelled with each expiratory movement. Dr. Bowles supports his statements by the recital of a number of cases which he has treated.

After the patient has been placed for a moment with face downward to allow the escape of water from the mouth and throat, he is turned on the side and *kept on that side* continuously, except when (about fifteen times a minute) the body is rolled for a few seconds upon the face again. By *keeping the same side always up* the lung on that side becomes clear. Turning first one and then the other side up is dangerous, because thereby the partly cleared lung is suddenly flooded with fluid from the lung which was downward. It is better to clear one lung entirely than to have both half cleared. Each time the body is turned upon the face a little more froth and water escapes from the mouth and nostrils. If one lung is thus cleared it may escape the inflammation which results from the inspiration of water. When the upper lung has been almost cleared, Dr. Bowles finds it useful to raise the upper arm above the head as in the Sylvester method, since the entrance of larger quantities of air into the lung is now safe. Pressure on the back at each pronation assists the escape of water somewhat, and it has a good influence on the heart, aiding the propulsion of blood towards the lungs. The continued use of the pronolateral method is an excellent mode of keeping the pharynx clear of obstruction.

